# Utilization of *Aloe* Compounds in Combatting Viral Diseases

**DOI:** 10.3390/ph15050599

**Published:** 2022-05-13

**Authors:** Erica Españo, Jiyeon Kim, Jeong-Ki Kim

**Affiliations:** Department of Pharmacy, Korea University College of Pharmacy, Sejong 30019, Korea; eespano@korea.ac.kr (E.E.); jiyeon17@korea.ac.kr (J.K.)

**Keywords:** *Aloe barbadensis*, *Aloe vera*, antivirals, adjuvants, immunomodulator, phytochemicals, aloe emodin, aloin, acemannan, natural products

## Abstract

Plants contain underutilized resources of compounds that can be employed to combat viral diseases. *Aloe vera* (L.) Burm. f. (syn. *Aloe barbadensis* Mill.) has a long history of use in traditional medicine, and *A. vera* extracts have been reported to possess a huge breadth of pharmacological activities. Here, we discuss the potential of *A. vera* compounds as antivirals and immunomodulators for the treatment of viral diseases. In particular, we highlight the use of aloe emodin and acemannan as lead compounds that should be considered for further development in the management and prevention of viral diseases. Given the immunomodulatory capacity of *A. vera* compounds, especially those found in *Aloe* gel, we also put forward the idea that these compounds should be considered as adjuvants for viral vaccines. Lastly, we present some of the current limitations to the clinical applications of compounds from *Aloe*, especially from *A. vera*.

## 1. Introduction

Recent large outbreaks of acute viral diseases, including those caused by the severe acute respiratory syndrome coronavirus (SARS-CoV)-2, Ebola virus, Zika virus, chikungunya virus, SARS-CoV, Middle Eastern respiratory syndrome CoV, and influenza virus, have underscored the limitations of currently available tools for controlling viral outbreaks. Ideally, both antivirals and vaccines should be on hand in the event of an outbreak; antivirals will be given to those with active infection, and vaccines will be administered to the larger population. However, as highlighted by the COVID-19 pandemic caused by SARS-CoV-2, the current response to viral (re)emergence is highly reactive: we only start searching for antivirals or design vaccines after viral emergence [1]. There is no repository of antivirals that can be dispatched for rapid outbreak response, hence the spike in death and hospitalizations throughout the COVID-19 pandemic. In the absence of effective antivirals and prior to vaccine approval, only non-pharmaceutical interventions could be imposed, and these measures have severely impacted global economy.

The increasing frequency of viral outbreaks necessitates global preparedness so that we may avoid a repeat of the protracted COVID-19 pandemic. Building a library of antivirals with broad-spectrum potential and developing them up to early human clinical trials is an ideal strategy in preparing for future outbreaks of known and unknown viral etiology. This strategy requires less knowledge of the emergent virus and, in turn, shorter lead time between outbreak and clinical deployment [1]. Further discovery and development of broad-spectrum antivirals and the expansion of targets of current antiviral candidates will therefore be instrumental to the management of future viral epidemics and pandemics [2]. 

Natural products contribute structural diversity and complexity to the current pool of drug candidates. Plants, in particular, are acknowledged as primary sources of new bioactive compounds. As of 2018, 44.1% of the reported natural compounds came from plants [3]. New plant species are continuously being discovered, adding further potential sources of novel bioactive compounds. However, despite their long history in traditional medicine and their success rates in other disease states, plants remain underutilized resources in the fight against viral diseases. Although secondary metabolites from plants have already been reported to inhibit viral infections in preclinical studies, an antiviral from plants is yet to be approved [4]. While synthetic small molecules, the most commonly approved antivirals, can be designed by chemists, the human imagination bears limitations. New ideas can be drawn from existing products in plants that have already evolved to be biologically active. Thus, plant compounds should be considered in developing new drugs, including those needed for the management and prevention of viral diseases.

*Aloe vera* (L.) Burm. f. (syn. *Aloe barbadensis* Mill.) is a succulent that grows in dry regions of Asia, Africa, America, and Europe. It belongs to the *Asphodelaceae* (*Liliaceae*) family, along with various *Aloe* species. The *A. vera* leaf consists of three layers that serve as sources of different compounds. The outermost layer or the rind, appears green and is the site of carbohydrate and protein synthesis [5,6]. The middle layer or the latex consists of yellow sap containing phenolics, including anthraquinones that are primarily present as glycosides. The innermost layer is the pulp, consisting of a clear gel that contains mainly polysaccharides such as acetylated mannans. *A. vera* extracts have long been used in folk medicine to cure digestive problems, skin problems, burns, wounds, diabetes, and high blood pressure [7]. It is also used as an additive in cosmetic products.

There is a large amount of evidence on the immunomodulatory effects compounds from *A. vera*, especially from *A. vera* gel. To a lesser extent, antiviral effects of *A. vera* extracts and compounds have also been reported. However, literature that weaves together the potential applicability of *A. vera* compounds in the management and prevention of a broad spectrum of viral diseases is limited. To fill this gap, here we discuss the potential of compounds isolated from *Aloe* spp., particularly *A. vera*, as direct-acting antivirals and as immunomodulators for the management of acute viral diseases. Additionally, we discuss the potential of some of the immunomodulators from *Aloe* as viral vaccine adjuvants to help prevent viral diseases. This manuscript should then provide information on *A. vera* compounds that can be developed to help combat the continuous threat of viral diseases.

## 2. Methodology

Literature searches for the antiviral effects of *Aloe* spp. and *Aloe* compounds were mainly performed using PubMed and Google Scholar employing “*Aloe* virus” or the name of the compound plus “virus” as search terms. For the immunomodulatory effects and adjuvant effects, “*Aloe* immune” and *“Aloe* adjuvant” were used as primary search words, with additional searches including specific targets such as “innate immunity,” “antibody,” or “T cell”. The results were then screened for their potential application in the context of viral diseases. In silico studies without in vitro data for the antiviral effects of the *Aloe* compounds were excluded from this review. References that attributed the effects of emodin to aloe emodin were also excluded under the premise that these isomers may possess different biological activities unless the primary source presented data for aloe emodin. The references used here were published from 1911 to 2022.

## 3. *Aloe* Compounds as Antivirals

### 3.1. Aloe Extracts Demonstrate Antiviral Activity

One of the earliest reports on the antiviral activity of *Aloe* extracts was a study that tested the effects of fractionated exudates (with and without ethanol treatment) from *A. vera* leaf gel against the human cytomegalovirus (HCMV) (Table 1) [8]. In the study, application of the exudates to HCMV-infected cells in the middle stages of the viral infection cycle yielded reduced HCMV plaque formation, suggesting that the extracts inhibited HCMV DNA synthesis.

Several studies on the antiviral effects of extracts from *A. vera* and from other *Aloe* species followed thereafter (Table 1). Exudate from the leaves of *A. secundiflora* was reported to reduce the mortality and severity of Newcastle disease in chickens [11]. Likewise, crude ethanol extracts from the leaves and flowers of *A. hijanzensis* reduced the egg infectivity of several hemagglutinating viruses, namely, Newcastle disease virus (NDV), a highly pathogenic strain of avian influenza virus (H5N1 subtype), egg drop syndrome virus, and avian paramyxovirus type 1 [14].

*A. vera* extracts have also exhibited dose-dependent inhibitory effects on herpes simplex virus 1 and 2 (HSV1 and HSV2) in vitro [9,10]. Interestingly, hot glycerin extract from *A. vera* gel was able to inhibit the pre-entry, entry, and post-attachment stages of HSV2 infection, suggesting multiple targets in the HSV2 replication cycle. Commercially prepared *A. vera* extract was also reported to inhibit coronavirus porcine epidemic diarrhea virus (PEDV) infection in vitro and in vivo [12]. Specifically, powdered *A. vera* extract inhibited PEDV replication in Vero and IPEC-J2 cells in a dose-dependent manner and appeared to target post-entry stages of infection, likely the genomic replication stage. The results of this study also indicated that components of the *A. vera* extract may be able to inactivate PEDV particles. Oral administration of the powdered *A. vera* extract also appeared to protect piglets from PEDV-induced intestinal pathology.

Crude *A. vera* extract and *Aloe* anthraquinones have also been tested for activity against murine norovirus 1 (MNV1) [13]. *A. vera* extract and, to a lesser extent, *Aloe* anthraquinones displayed dose-dependent inhibitory effects on MNV1. Pretreatment of MNV1 particles with *A. vera* extract was found to be highly effective against the virus, suggesting that the extract affects early stages of infection. *A. vera* applied on fresh food surfaces was also able to reduce the infectivity of MNV1, indicating that components of *A. vera* may be used to treat food to prevent food-borne viral infections. 

Biaron C, a medicinal product containing aqueous extracts from *A. arborescens* is used in Poland, Russia, and Ukraine, especially for children with upper respiratory tract infections [16]. Biaron C has been reported to reduce human rhinovirus (HRV14) production in HeLa cells in a dose-dependent manner [15]. This study also showed that Biaron C inhibited other viruses that infect the human respiratory tract, including coxsackievirus A9, influenza A (H1N1 and H3N2) viruses, and influenza B (Yamagata and Beiying) viruses. It also showed modest inhibitory effects on parainfluenza virus 3 and respiratory syncytial virus infection. However, Biaron C failed to inhibit adenovirus 5 infection in vitro. A previous review has summarized the findings of a number of clinical trials and post-marketing studies on Biaron C [16]. These small observational studies suggest benefits of Biaron C to children (3–12 years) with recurrent or chronic upper respiratory tract infections, as indicated by decreased recurrence and accelerated recovery, with few mild and often self-clearing adverse effects.

Evidently, extracts from different species of *Aloe* have the capacity to inhibit several types of animal and human viruses. However, crude extracts are complex mixtures of bioactive compounds and are often highly variable due to differences in plant source and extraction procedures. The identification of one or two active components would therefore be more beneficial for clinical application, standardized production, and mechanistic studies. In the following sections, we discuss some of the active compounds in *Aloe* spp., especially those in *A. vera*, that exhibit the most promise as antivirals.

### 3.2. Antiviral Activities of Phenolic Compounds from A. vera

#### 3.2.1. Aloe Emodin

**Figure 1:**
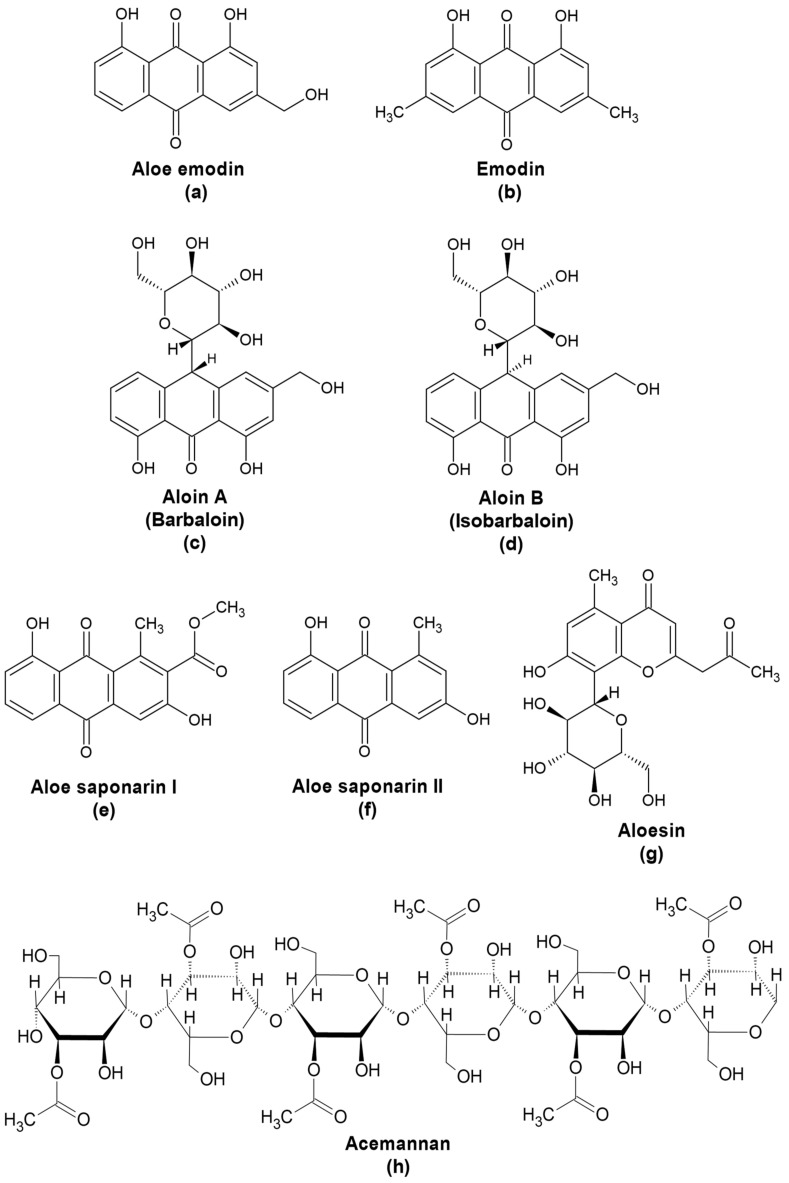
Compounds from *Aloe* spp. with antiviral and immunomodulatory activities. (**a**) Aloe emodin, an anthraquinone found in *Aloe* latex is an isomer of (**b**) emodin, which exhibits antiviral activity. Aloins (**c**) A and (**d**) B are bitter yellow anthraquinone glycosides in the *Aloe* latex and can be used to synthesize aloe emodin. Alosaponarins (**e**) I and (**f**) II are also anthraquinones found in the latex of *Aloe* species. (**g**) Aloesin is a chromone from the latex of various *Aloe* species. (**h**) Acemannan or acetylated mannan is the predominant polysaccharide found in *Aloe vera* gel.

As with most plants, *Aloe* is rich in secondary metabolites especially anthraquinones. Aloe emodin (1,8-dihydroxy-3-(hydroxymethyl)-9,10-anthracenedione; AEM), an isomer of emodin (Figure 1), was first isolated from rhubarb (*Rheum rhaponticum*) in 1913 [17]. It has since been identified in several plants including *A. vera*, *Rhamnus frangula*, *Rhamnus purshiana*, and *Cassia angustifolia*. In *Aloe*, AEM is typically present in extracts from the gel, sap, or leaves. Similar to emodin, AEM exhibits a number of pharmaceutical effects, including antiviral, anticancer, anti-inflammatory, antibacterial, and anti-parasitic activities. Although AEM and emodin have similar pharmaceutical effects, they possess different potencies toward their targets and may even affect different pathways [18]. Thus, although emodin is a highly reported antiviral, its antiviral activity and targets may not necessarily translate to AEM [19].

A seminal study performed by Sydiskis et al. showed that AEM reduced the infectivity of several enveloped viruses, namely, HSV1, HSV2, varicella-zoster virus (VZV), pseudorabies virus, and influenza A virus (IAV) without apparent cytotoxic effects in vitro (Table 2) [9]. Transmission electron microscopy revealed that preincubation with anthraquinones, including AEM, disrupted the HSV1 envelope, suggesting that AEM may be used against enveloped viruses to destroy the lipid envelope. Meanwhile, Lin et al. have demonstrated that AEM exhibits inhibitory effects on enterovirus 71, a non-enveloped virus, in vitro, suggesting that AEM has antiviral effects on both enveloped and non-enveloped viruses [20]. Lin et al. also showed that AEM inhibits Japanese encephalitis virus (JEV) infection in vitro. A different study has shown that AEM inhibits the protease activity of the JEV NS2B-NS3A protein (IC_50_ of 7.3 μg/mL), suggesting a mechanism underlying the anti-JEV effects of AEM [21].

AEM has also displayed inhibitory effects on CoVs. A study has shown that AEM inhibits the activity of the 3C-like protease of SARS-CoV in a dose-dependent manner [22]. A molecular docking study has also suggested that AEM binds N-terminal RNA-binding domain of the SARS-CoV-2 nucleocapsid protein [31]. These indicate the potential of AEM as an agent for the treatment of diseases caused by human CoVs. 

Moreover, AEM has been reported to reduce the production of S and E antigens in HepG2.2.15 cells infected with hepatitis B virus (HBV), indicating that AEM inhibits HBV infection [23]. Remarkably, the effects of AEM were comparable to those of lamivudine, a nucleoside analogue used for the management of HBV and HIV infection. The in vitro study also showed that AEM enhances the effects of lamivudine, suggesting a potential for combinatorial therapy. In silico analyses further suggested that AEM may inhibit HBV replication by binding the HBV polymerase.

The antiviral effects of AEM seem to apply to a broad range of viruses: RNA and DNA, enveloped and non-enveloped, indicating its potential as a broad-spectrum antiviral. However, whether it has similar targets across related viruses remains to be determined.

AEM has poor intestinal absorption, a short half-life, and low bioavailability, making it unideal for oral administration [32]. As with emodin, AEM is a stimulant laxative, which may not be an acceptable side effect for clinical application. Furthermore, AEM has been reported to be phototoxic, nephrotoxic, and hepatotoxic in animal models [32]. The derivatization of AEM is currently being investigated to improve its pharmacological and safety profiles [33].

#### 3.2.2. Aloin

Aloin A (barbaloin; (10-beta-D-glucopyranosyl-1, 8-dihydroxy-3-hydroxymethyl-9(10H)-anthracenone)) and its diastereoisomer aloin B (isobarbaloin) (Figure 1), are commonly found in *A. vera* latex. Oxidative cleavage of the glycosidic linkage in both types of aloin yields AEM. Because *Aloe* extracts generally contain higher amounts of aloin (18–25%, reaching up to 30% of dry weight) than AEM, aloin from *A. vera* can be used to synthesize AEM [7,34].

Aloin itself also exhibits antiviral activity (Table 2). It displays inhibitory effects on both oseltamivir-sensitive and oseltamivir-resistant strains of influenza A H1N1 virus in vitro, likely through the inhibition of the viral neuraminidase (NA) [24]. Notably, aloin also inhibited influenza A H3N2 virus and influenza B virus, which is generally less susceptible to oseltamivir treatment. In the same study, aloin was also shown to increase the survival of mice against lethal challenge with the influenza A H1N1 (PR8) virus, with evidence of faster viral clearance relative to untreated mice. Further, hepatic and renal toxicity tests did not show any safety signal for aloin treatment (2.5 mg/kg body weight) in mice. Moreover, both isoforms of aloin have been demonstrated to inhibit the proteolytic and deubiquitinating activities of the papain-like protease of SARS-CoV-2 [35].

Similar to AEM, isobarbaloin was reported to reduce the production of HB S and E antigens in vitro [23]. However, the effects of AEM were superior to those of isobarbaloin. Remarkably, barbaloin-loaded liposomes inhibited viral hemorrhagic septicemia rhabdovirus infection of epithelioma papulosum cyprinid cells, especially at early stages of infection [25]. Meanwhile, free barbaloin exhibited no such effects, suggesting that barbaloin integrates into phospholipid membranes and that loading barbaloin into liposomes allows barbaloin to disrupt the viral envelope. Further studies on the effects of barbaloin on other enveloped viruses are needed to support this hypothesis. At the time of writing, there has been no report on the effects of barbaloin on non-enveloped viruses.

A study has suggested that aloin is rapidly absorbed in the gut of a rat model and that low concentrations may be adequate to reach desired pharmacological effects [36]. This same study also showed that isobarbaloin may be excreted more slowly than barbaloin in rats, potentially making it more effective in this model. It should be noted, however, that certain human gut bacteria can convert barbaloin to AEM, which may alter the pharmacological profile of barbaloin in humans [37]. Further, reports on the toxicity of aloin have led to the regulation of maximum allowable aloin content of products with *A. vera*-derived ingredients (10 ppm for oral consumption) [38,39]. Whether this impacts its clinical applicability should be investigated. Optimization of aloin to improve its safety profile should be considered.

#### 3.2.3. Other Phenolic Compounds from *Aloe*

The anthraquinones aloesaponarins I and II (Figure 1) are found in different *Aloe* species. Aloesaponarin II was reported to reduce cytopathic effects induced by oseltamivir-susceptible but not by oseltamivir-resistant IAV [26]. In contrast, aloesaponarin I did not exhibit inhibitory effects on IAV infection in vitro. Interestingly, derivatives of both aloesaponarin I and II reduced IAV production in vitro especially when added at late stages of infection. Good yield was reported for the synthesis of one of the derivatives (88%). Altogether, these findings suggest that derivatization of aloesaponarins should be considered an approach for maximizing the benefits of these anthraquinones from *Aloe*.

### 3.3. Polysaccharides from Aloe

Polysaccharides, including mannans, galactans, and pectins, comprise around 53% of the dry weight of *A. vera* gel [40]. Much of the reported pharmaceutical activities of *Aloe* are attributed to the polysaccharides in the gel taken from the pulp, which also includes cell walls and degenerated organelles [41]. Because of this, although the term “gel” is often used interchangeably with “pulp”, not all reported gel extracts are purely from the gel component.

A group has reported that an *Aloe* polysaccharide (APS) fraction inhibited influenza A H1N1 (PR8) virus infection in Madin–Darby canine kidney cells (Table 2) [27]. These results were corroborated by their in vivo findings, wherein APS, especially at a high dose (40 mg/day), protected mice from pathology induced by infection with influenza A H1N1 (PR8) virus. Specifically, APS treatment alleviated lung injury caused by influenza virus infection and reduced lung viral titers. Notably, addition of APS at the pre-infection and adsorption stages of viral infection in vitro appeared optimal, suggesting that components of the APS fraction may act directly on the viral particles thereby reducing its infectivity. Transmission electron micrographs of APS-treated viral particles revealed irregularly shaped viral particles with low or no-electron density cores around APS clusters. This suggests that APS inhibits IAV infection through physical destruction of the viral particles.

#### Acemannan as an Antiviral

Acemannan (ACM) has been adopted as the term to refer to mannans isolated from *A. vera*, specifically from the leaf gel. ACM pertains to long-chain polydispersed β(1,4)-acetylated mannans (Figure 1) in *Aloe* and has been associated with various pharmaceutical effects. ACM has been reported to inhibit HIV-1 replication in a dose-dependent manner in vitro and was hypothesized to modify glycosylation of the HIV-1 glycoprotein (Table 2) [28]. Further, the combination of ACM with suboptimal concentrations of azithymidine inhibited HIV-1 production. The combination of ACM with acyclovir also appeared to be synergistic in vitro. Moreover, there are reports on the clinical stabilization of cats with feline leukemia virus following ACM treatment and on the potential benefits of ACM to cats infected with feline immunodeficiency virus [29,30]. However, whether the effects of ACM on these cats are due to the immunomodulatory activity of ACM instead of or in addition to direct antiviral effects remains to be determined.

## 4. *Aloe* Compounds for the Regulation of Immune Responses to Viral Infection

**Figure 2:**
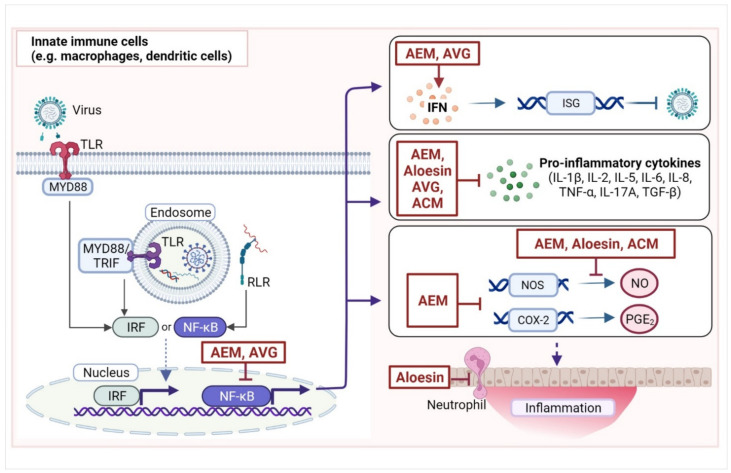
Targets of compounds from *Aloe*, especially *Aloe vera*, in the innate immune response. Innate immune cells, including macrophages and dendritic cells, recognize viral infection through pattern recognition receptors such as the Toll-like receptors (TLR3, TLR7, TLR8, and TLR9) or RIG-I-like receptors (RLR). Upon signal stimulation, TLRs recruit adaptor proteins, such as myeloid differentiation primary response 88 (MYD88) and TIR domain-containing adapter molecule 1 (TRIF). This initiates signaling cascades that activate transcription factors, mainly interferon regulatory factors (IRFs) and nuclear factor kappa B (NF-κB). IRFs are typically involved in the production of interferons (IFNs) that, in turn, stimulate the expression of products of IFN-stimulating genes (ISGs) that have antiviral functions. Meanwhile, NF-κB is a master regulator of the immune response and activates the transcription of several proteins involved in the immune response including ISGs and cytokines, such as interleukins (IL), tumor necrosis factor α (TNF-α), and transforming growth factor β (TGF-β). NF-κB also activates the transcription of the inducible nitric oxide synthase (NOS)-2 gene, which catalyzes the production of nitric oxide (NO) that regulates local inflammation. Moreover, NF-κB activates the transcription of cyclooxygenase-2 (COX-2), which converts arachidonic acid to prostaglandin E_2_ (PGE_2_), which induces inflammation. Tissue inflammation recruits other cells, such as granulocytic neutrophils, to the site of infection. Shown here are some of the reported targets of compounds from *Aloe* spp., especially *A. vera*, such as aloe emodin (AEM), *A. vera* gel (AVG), acemannan (ACM), and aloesin in the innate immune response. While in most cases, *A. vera* compounds inhibit pro-inflammatory responses, they may also promote certain responses, such as IFN production. (Image was created with BioRender.com, accessed on 27 April 2022).

Viruses that penetrate the initial line of defenses (skin and mucosa) and enter the host are detected by pattern recognition receptors, especially Toll-like receptors (TLR3, TLR7, TLR8, and TLR9), retinoic acid-inducible gene I, and NOD-like receptors that are typically expressed in innate immune cells (e.g., dendritic cells and macrophages) (Figure 2) [42]. Upon activation, these receptors trigger the production of interferons (IFNs); chemokines; and pro-inflammatory cytokines, such as interleukins (IL-2, IL-6, IL-8, IL-1β, IL-12, IL-17) and tumor necrosis factor (TNF-α) via transcription factors nuclear factor kappa B (NF-κB) or IFN regulatory factors [43]. The IFNs then activate IFN-stimulated genes (ISGs), which exhibit various antiviral activities. Meanwhile, pro-inflammatory cytokines promote local inflammatory responses (e.g., vasodilation, vascular permeability, and tissue destruction) and the recruitment of innate effector cells (e.g., neutrophils, natural killer cells, and innate lymphoid cells) to facilitate the elimination of virus-infected cells. During this process, prostaglandins, especially prostaglandin E_2_ (PGE_2_) produced from arachidonic acid by cyclooxygenase-2 (COX-2), cause some of the classical signs of inflammation (redness, swelling, pain, and heat) in affected tissues. PGE_2_ is also involved in the regulation of cytokine production in macrophages and dendritic cells (DCs).

Activated DCs at the site of infection migrate to lymphoid tissues to present antigens to T cells, thereby initiating the adaptive immune response [44]. Viral antigen presentation by DCs stimulates the differentiation of naïve CD8^+^ T cells to cytotoxic T cells (CTLs) that destroy virus-infected cells. It also activates CD4^+^ T cells, which differentiate into T helper 1 (T_H_1) cells that further produce antiviral cytokines, and into T follicular helper (T_FH_) cells that promote antibody production in B cells. Adequate viral clearance typically allows the resolution of inflammation and a return to homeostasis. DCs, macrophages, activated regulatory T cells, B cells, and some natural killer cells secrete the anti-inflammatory cytokines IL-10 and transforming growth factor (TGF)-β, which can then block the production of pro-inflammatory cytokines in different target cells [43]. 

A persistent and exaggerated inflammatory response can cause tissue and organ damage that is seen in severe cases of acute viral diseases. Acute respiratory distress syndrome following COVID-19 or influenza virus pneumonia are characterized by persistent infiltration of immune cells in the lungs and by dysregulated production of pro-inflammatory cytokines, which eventually lead to organ damage [45]. On the other hand, inadequate immune responses, such as a failure or delay in producing IFNs, can also lead to the inability to clear the virus and a failure in initiating the cascade of the necessary immune responses [46,47]. Thus, modulating immune responses following viral infection may prove helpful in treating diseases. That corticosteroids were among the first drugs to show benefits for the management of severe COVID-19 suggests that immune regulation should be considered for the treatment of viral diseases with similar hyper-immunological profiles. *A. vera* extracts have demonstrated anti-inflammatory activity in several studies. In the following sections, we discuss some of the anti-inflammatory effects of *Aloe* compounds and the potential mechanisms underlying these effects.

### 4.1. Aloe Phenolics as Immunomodulatory Agents

#### 4.1.1. Immunomodulatory Effects of Aloe Emodin

AEM has also been reported to exhibit anti-inflammatory effects in vitro and in vivo (Table 3) (Figure 2). In a study by Yu et al., AEM was found to reduce the activity of natural killer cells and the phagocytic activity of macrophages from rats in a dose-dependent manner. Moreover, AEM was found to increase IL-1β and tumor necrosis factor (TNF)-α but not IL-6 and IFN-γ production of leukocytes [48]. Further supporting the anti-inflammatory effects of AEM on macrophages, incubation of RAW 264.7 macrophages with AEM attenuated lipopolysaccharide (LPS)-induced upregulation of inducible nitric oxide synthase (*Nos*) mRNA and nitric oxide (NO) production [49]. AEM was also found to reduce PGE_2_ production in LPS-stimulated macrophages. Further, AEM inhibited LPS-induced *Cox2* mRNA levels in a dose-dependent manner in the macrophages. A rat paw edema model also indicated the anti-inflammatory effects of AEM and a derivative [50]. Moreover, in rats with complete Freund’s adjuvant-induced arthritis, AEM and a derivative reduced NO production in the rats relative to the untreated control. NO acts as an inflammatory mediator, and can be either anti- or pro-inflammatory, depending on concentration. Modulating NO production is therefore important in regulating the inflammatory response.

In a middle cerebral occlusion reperfusion rat model, AEM was able to reduce serum TNF-α levels, and protected rats from neurological deficits, suggesting that AEM protected the rats from neuroinflammation [51]. AEM treatment of LPS-stimulated microglial BV2 cell lines was able to inhibit the production of NO as well as pro-inflammatory cytokines IL-6 and TNF-α. In this model, AEM reduced the activity of NF-κB in a dose-dependent manner. Considering that NF-κB is a master regulator of immune responses, activating the transcription of ISGs, cytokines, NOS, and COX-2, the anti-inflammatory effects of AEM is likely mediated by NF-κB (Figure 2).

In the context of viral infections, AEM was able to stimulate the activation of IFN-α transcription in vitro [20]. It also activated the IFN stimulation response element (ISRE)-driven promoter and the gamma-activated sequence (GAS)-containing promoter, which are involved in the expression of ISGs. Indeed, AEM enhanced the transcription of two ISG products, protein kinase R (PKR) and 2′,5′-oligoadenylate synthetase (OAS), in a monocytic cell line (HL-CZ cells). Thus, AEM may be able to inhibit viral infection by enhancing the antiviral immune state. 

Supporting this, Li et al. [52] demonstrated that the inhibitory effects of AEM against IAV correlated with increased levels of galectin-3, which, in turn, upregulated IFN-β and IFN-γ. The non-structural protein 1 (NS1) of influenza A viruses inhibits the IFN-α-inducible Janus kinase-signal transducer and activator of transcription (JAK-STAT) signaling pathway that typically regulates the expression of ISGs [66]. In their study, Li et al. further showed that AEM was able to attenuate the effects of NS1 in vitro, promoting the phosphorylation of STAT1, which in turn promoted the expression of PKR and OAS in NS1- producing cells. However, the effects of AEM may be independent of IFN-α, as IFN-α did not increase in response to AEM treatment. Meanwhile, galectin-3 has been suggested to activate the JAK-STAT pathway [67]. Thus, the findings of Li et al. suggest that AEM activates the JAK-STAT pathway through galectin-3. However, these results will have to be replicated and characterized.

Thus, in addition to direct antiviral effects, AEM appears to modulate immune responses, including those that are relevant to viral infections. An antiviral agent with the capacity to target both viral and host factors would prove beneficial especially in combatting viruses that tend to develop resistance against direct-acting antivirals, as is the case for IAV and HIV drugs. Furthermore, an antiviral agent with immunomodulating capacity can be administered in a large therapeutic window over the course of a viral disease. Antivirals are given early in the course of the disease to accelerate viral clearance, while immunomodulators are given late in the disease course, typically in severe disease cases, to temper hyper-immune responses. A drug that targets both the virus and the immune response may be given in the middle stages of infection to reduce the likelihood of progression to severe or critical illness.

#### 4.1.2. Immunomodulatory Effects of Aloin

In addition to inhibitory effects on influenza virus infection in vitro, aloin also exhibits immunomodulatory effects on influenza A H1N1 (PR8)-infected mice with adoptively transferred hemagglutinin (HA)-specific CD4^+^ and CD8^+^ T cells [24]. Pulmonary infiltration of CD4^+^ and CD8^+^ T cells increased following aloin treatment in infected mice, and these cells showed increased production of IFN-γ and TNF-α. NA-mediated TGF-β activation was also reduced in the lungs. Co-treatment with aloin also appeared to enhance the effects of oseltamivir on HA-specific T cell responses in influenza-infected mice. Specifically, aloin augmented the oseltamivir-induced reduction in TGF-β and increase in IFN-γ. Similar trends were observed when infected mice were first treated with aloin and then treated with oseltamivir two days later. 

Influenza NA activates TGF-β by removing sialic acid motifs from the latent form of TGF-β. This cytokine has both pro- and anti-inflammatory functions. Inhibition of TGF-β has been observed to reduce epithelial cell adherence of group A *Streptococcus* bacteria, which are common coinfections with influenza virus that contribute to the morbidity and mortality of influenza virus infections [68]. TGF-β may also act as a pro-viral factor in influenza virus infection by suppressing the production of IFN-β, which allows viral proliferation, further indicating that increased levels of TGF-β may augment the pathology of influenza virus infection [69]. Meanwhile, increased TNF-α production has been correlated with reduced viral titers in vitro, suggesting that it has antiviral functions [70]. Thus, the ability of aloin to inhibit TGF-β and to promote TNF-α may contribute to the protective effects of aloin in influenza A H1N1 (PR8)-infected mice. However, the immune pathways directly targeted by aloin in the context of influenza virus infection will have to be elucidated further.

#### 4.1.3. Anti-Inflammatory Effects of Aloesin

Aloesin is a chromone (Figure 1) found in the latex of a number of *Aloe* species, and it has exhibited anti-inflammatory activity. An aloesin-supplemented diet markedly reduced deformity and granulocyte infiltration in colon segments taken from rat colitis models, indicating anti-inflammatory effects of aloesin (Table 3) [53]. Serum TNF-α and PGE2 levels, and colonic *Tnfa* and *Il1b* mRNA levels were also reduced by supplementation of aloin, *Aloe* gel, or aloesin to the diets of the rat colitis models. Moreover, rats fed with aloin, *Aloe* gel, or aloesin had reduced plasma levels of leukotriene B4 (LTB_4_), an immunomodulatory chemokine, with aloesin reducing LTB_4_ levels to baseline. LTB_4_ is involved in the recruitment of innate immune cells, which leads to the production of cytokines [71]. LTB_4_ may be one of the direct targets of aloesin, leading to overall changes in cytokine levels. However, a chromone analog was also indicated to reduce TNF-α production through the inhibition of NF-κB, suggesting that NF-κB may also be a target of aloesin [72].

### 4.2. Immunomodulatory Effects of A. vera Gel and Its Components

#### 4.2.1. Immunomodulatory Effects of *A. vera* Gel

Most of the anti-inflammatory and immunomodulatory effects of *Aloe* extracts are attributed to the gel and polysaccharides in the gel. In a study by Langmead et al., incubation with *A. vera* gel (AVG) of mucosal biopsies from patients with active ulcerative colitis reduced PGE_2_ release from the biopsies (Table 3) [54]. Additionally, they showed that AVG reduced IL-8 production in human colorectal cancer (Caco-2) cells at a select concentration, suggesting that AVG has anti-inflammatory effects. AVG was also able to reduce the production of cytokines TNF-α, TGF-β, and IL-6 in rats given a high-fat diet [55]. Furthermore, rats with acetic acid-induced gastric ulcer had lower serum TNF-α levels, and higher IL-10 levels compared to the untreated ulcer control. Gastric ulcer also resolved faster among the *A. vera*-fed rats than among the untreated rats. 

Moreover, AVG was found to reduce LPS-induced cytokine (IL-6, IL-8, IL-1β, and TNF-α) production in THP-1 cells and in primary human monocyte-derived macrophages [56]. Further investigation of underlying mechanisms for the effects of AVG on IL-1β suggested that AVG downregulated both pro-IL-1β and IL-1β in LPS-stimulated primary macrophages but did not appear to directly affect pro-IL-1β and IL-1β levels in the absence of LPS, suggesting that AVG only suppresses inflammation in the presence of stimulation and does not affect the baseline state. The effects of AVG on IL-1β may also be related to the downregulation of the NOD-, LRR- and pyrin domain-containing protein 3 (NLRP3) sensor protein and the P2X7 receptor, which are both involved in the activation of the NLRP3 inflammasome. Notably AVG inhibited LPS-induced activation of NF-κB and kinases in the MAPK pathway, which are both involved in the recognition of LPS by TLR4. These findings suggest that the attenuation of LPS-induced increases in IL-1β and other pro-inflammatory cytokines by AVG may be mediated by the NF-κB or the MAPK pathway. If, indeed, NF-κB is affected by AVG, then downstream inflammatory responses (Figure 2) may be affected. 

Several in vivo studies further support AVG-induced reduction of pro-inflammatory cytokines (TNF-α, IL-6, IL-1β, IL-4, IFN-γ, and IL-17A) in the presence of various stimuli (e.g., LPS, bacteria, and pesticides) or in different inflammatory conditions (e.g., atopic dermatitis, arthritis, and ulcer in rodents) (Table 3) [57,58,59,73,74]. Interestingly, some studies also reported increased production of IL-10, which is involved in the resolution of inflammation [60,73]. Thus, AVG may not only attenuate inflammation through the reduction of pro-inflammatory cytokines but may also accelerate the shift from the pro-inflammatory immune state to a recovering immune state.

Freeze-dried *A. vera* inner leaf gel (AVH200) reduced the activation and proliferation of T cells in a dose-dependent manner [61]. However, it did not trigger significant apoptosis among activated T cells, suggesting that activation of the apoptotic pathway is not the underlying mechanism for the effects of AVH200 on T cells. AVH200 also reduced cytokine secretion (IL-2, IL-5, and IL-17A) in T cells, which may be a consequence of reduced T cell proliferation. Additionally, the effects of the inner leaf gel on T cell proliferation were superior to the effects of *A. vera* extract, indicating that the active component is predominantly in the inner leaf gel. Notably, *A. vera* extract alleviated symptoms of inflammatory bowel syndrome in two small randomized-controlled studies, further indicating the anti-inflammatory effects of *A. vera* components [75].

Taken together, AVG appears to have immunosuppressive effects in the presence of stimuli or in the inflammatory disease state, suggesting a potential for application in viral diseases characterized by hyper-inflammatory responses. The mechanisms and pathways involved in the immunomodulatory effects of AVG and their direct effects on the viral disease state will have to be determined in future studies.

#### 4.2.2. Immunomodulatory Effects of Acemannan and *A. vera* Polysaccharides

As opposed to the observed immunosuppressive effects of AVG, a number of immunostimulatory effects have been reported for ACM (Table 3) (Figure 2). Womble and Helderman reported that ACM promoted CTL genesis and improved their capacity to destroy target cells [62]. ACM also enhanced monocyte activity in response to alloantigen, which may contribute to the CTL antiviral response. Moreover, ACM was reported to enhance macrophage activation in response to IFN-γ stimulation, which may indicate a favorable antiviral immune response [63]. 

In the absence of stimulation, ACM was also able to induce cytokine (IL-6 and TNF-α) and NO release in macrophages. Similarly, ACM activated NO production in chicken spleen cells and chicken macrophages, with indications that the effects may be mediated by a receptor for terminal mannose [64]. This was further supported by the ability of modified APS, especially those within the 5–400 kDa range, to induce the maturation of RAW 264.7 cells [76]. Modified APS also promoted the production of TNF-α, IL-1β, and NO in the macrophages. In contrast, however, a separate group reported that the combination of ACM and IFN-γ caused NO-independent apoptosis in RAW 264.7 macrophages, and that the combination downregulated B-cell lymphoma 2 (*Bcl2*) at the mRNA level [77]. The mechanisms underlying the effects of ACM on NO production remain unclear and will have to be elucidated further. Regardless, macrophages are involved in the clearance of virus-infected cells. Thus, ACM may promote the antiviral immune state.

Moreover, ACM was discovered to be mitogenic to mouse splenocytes [65]. It also stimulated the differentiation and maturation of DCs from mouse blood monocytes. However, mannose did not inhibit the effects of ACM on DCs, suggesting that these effects did not involve mannose-binding receptors.

Some groups have put forward the idea that components of *Aloe* gel, including polysaccharides, may exhibit either immunostimulatory or immunosuppressive effects, likely depending on purity, size, composition, or concentration of the gel or the compound [76,78]. Further studies using purified and characterized APS and ACM will have to be performed to elucidate the specific immunomodulatory activity of each compound. Additionally, based on the studies we presented here, the immune-related pathways targeted by ACM are so far unknown. More mechanistic studies on the immunomodulatory effects of ACM especially in the context of viral diseases will have to be performed so that its immunomodulating capacity can be maximized for clinical application.

#### 4.2.3. *Aloe* Compounds as Viral Vaccine Adjuvants

Adjuvants are additional vaccine components that enhance the magnitude, breadth, and durability of immune responses to vaccines. Although a number of adjuvants are licensed for use with certain vaccines, safer alternatives that stimulate antiviral immune responses are still desired. For instance, alum, the most commonly used adjuvant in human viral vaccines elicits a T_H_2-biased immune response needed to manage helminthic parasites instead of stimulating T_H_1 immune responses required to combat most viral infections. Given the immunomodulatory effects of *Aloe* components, especially *Aloe* polysaccharides such as ACM, *Aloe* compounds may be considered as adjuvants for viral vaccines (Table 4).

The viral vaccine adjuvant potential of ACM was first reported by Chinnah wherein subcutaneous administration of ACM with a combination vaccine for NDV vaccine, infectious bursal disease virus (IBDV), and infectious bronchitis in chickens raised protective antibody titers to NDV [79]. However, no enhancement of humoral immune responses to IBDV was observed, indicating that the adjuvanticity of ACM is antigen-dependent and ACM cannot be used as vaccine adjuvant for all viruses. Oral administration of AVG was reported to promote anti-sheep red blood cell antibody production in mice [85]. ACM has also been shown to raise anti-coxsackievirus B3 (CVB3) antibody titers in mice, although it did not ameliorate myocarditis arising from CVB3 infection [80]. Oral administration of processed *Aloe* gel (PAG) in mice also elevated HA antibody and virus neutralizing titers against homologous and heterologous IAV strains [81]. Moreover, oral PAG enhanced the protectivity of IAV antigen and vaccines against homologous and heterologous challenge, although the effects were not as dramatic as those of commercially available adjuvants. High levels of neutralizing antibody titers are considered correlates of immunity and protectivity for vaccines against several viruses [86]. The ability of AVG or ACM to enhance the humoral immune response indicate an enhancement of immune responses that may be relevant to vaccine protectivity, suggesting their potential as adjuvants.

Furthermore, *A. vera* compounds have been reported to stimulate cell-mediated immunity and influence the direction of the T cell immune response. In addition to increasing IgA, IgG, and IgM levels following vaccination against myxomatosis virus in rabbits, oral administration of APS also increased CD4^+^ and CD8^+^ T cell counts in vaccinated rabbits [82]. Notably, the administration of the combination of AVG and alum with a human papillomavirus (HPV16 E7d) vaccine increased the levels of T_H_1 cytokines IFN-γ and IL-4 in mice [83]. AVG alone (without vaccine) also increased the production IFN-γ and IL-4. Furthermore, AVG enhanced the effects of Montanide, an adjuvant known to enhance T_H_1 immune responses to vaccines, as indicated by elevated IgG2 titers following vaccination with HPV16 E7d. Although the study did not show the effects of AVG alone with the vaccine, the findings of this study suggest that adding AVG to an existing adjuvanted vaccine promotes a shift to a T_H_1 immune response, which is important in controlling viral infections. Whether the combination of AVG with an unadjuvanted viral vaccine promotes the T_H_1 immune response should be investigated further.

Although a number of animal studies indicate the viral vaccine adjuvant potential of AVG, we only know of one exploratory trial in humans so far. Oral intake of PAG (8 weeks) that overlapped with quadrivalent influenza vaccination (week 4) did not significantly increase vaccine seroprotectivity among healthy adults [84]. However, the geometric mean fold increase in antibody titers among patients at 4 weeks post-vaccination relative to baseline was significantly higher in the PAG group than in the placebo group. Furthermore, patients in the PAG arm reported lower rates of upper respiratory tract infection than those in the placebo arm, indicating that PAG may have increased the protectivity of the quadrivalent vaccine against symptomatic influenza infection. Cell-mediated immunity following influenza virus vaccination has been correlated with disease protection and cross-protection [87]; however, markers for cellular immunity were not evaluated in this study. The effects of PAG on the cell-mediated immunity conferred by the quadrivalent influenza vaccine will have to be evaluated to provide a clear understanding of the adjuvant potential of PAG. Additionally, the optimal route of administration and dose of PAG or its components for human applications require further investigation.

Taken together, compounds from *A. vera* have the ability to enhance the immunity and protectivity conferred by viral vaccines. The breadth of the adjuvant activity (e.g., target viruses) of the *A. vera* compounds should be determined in vivo.

## 5. Hurdles to Applying *Aloe* Compounds in Preventing and Managing Viral Diseases

As with other natural products, the clinical applicability of compounds from *Aloe* is complicated by poor product standardization and limitations to industrial-scale production. For example, the polysaccharide and phenolic content of *Aloe* spp. are highly variable and are easily affected by both biotic and abiotic factors [3]. Compound compositions of *Aloe* spp. generally differ based geographic origin, temperatures, climate, and variant, making harvest from direct plant sources difficult to replicate across multiple locations and various seasons [88]. The composition of the extracts and the sizes of polysaccharides are further affected by differences in treatment and extraction procedures. Moreover, even when farming, harvesting, and extraction conditions have been standardized, industrial-scale production of some of the compounds is often not feasible if plants are used as direct sources owing to low yield from individual plants [89]. Full synthesis, semi-synthesis, or bioengineering may be needed to achieve the desired amounts of compounds, especially the phenolics, for wide-scale clinical application. This requires research legwork to determine which metabolic pathways in microorganisms can be altered to produce the desired compound.

Another hurdle is the low bioavailability of *Aloe* phenolics, especially AEM and aloin. *Aloe* anthraquinones have also been reported to exhibit toxic effects in vitro and in vivo. These problems can be solved by optimization of the core structure of the compounds to improve their pharmacological and safety profiles. Compounds with low bioavailability can also be encapsulated using lipid-based delivery systems.

Lastly, most of the work we have presented here are still at early stages of pre-clinical exploration. For antiviral effects in particular, most studies on *Aloe* compounds remain in vitro and have little information on targets in viral replication cycles. Meanwhile, in the case of *Aloe* polysaccharides, the composition of *Aloe* polysaccharides (e.g., size range) are not well-defined such that different studies report contradicting results [81]. Characterization of isolated *Aloe* compounds and more mechanistic studies are needed to understand how the pharmacological activities of *Aloe* compounds can be maximized for application in viral disease prevention and management.

## 6. Conclusions

Here, we presented evidence that various compounds from *Aloe*, especially from *A. vera*, can be used as (1) direct antivirals for viral clearance; (2) immunomodulators for late viral disease management; and (3) vaccine adjuvants for viral disease prevention. However, most of these studies are still at early exploration stages. More characterization, mechanistic, and in vivo studies in the context of viral diseases are needed to understand the full potential of compounds from *Aloe*. Additionally, some of the *Aloe* compounds, especially the phenolics, are subject to the same issues as phenolics from other plants: low bioavailability and toxicity, which limit their clinical applicability. Optimization of these compounds should be considered. Furthermore, standardization of compound extraction procedures especially for *Aloe* polysaccharides will have to be ensured for industrial production. Overall, more research is needed to see these compounds further along the drug development pipeline. However, despite these issues, *Aloe* compounds contribute chemical and structural diversity to the current repertoire of candidate compounds for the fight against viral diseases. With the increasing frequency of viral disease emergence, all resources, whether natural or synthetic, should be given weight and developed pharmaceutically to improve our readiness for future viral outbreaks.

## Figures and Tables

**Table 1:** Antiviral activities of extracts from *Aloe* spp.

Source	Extract	Virus	Observation/Target	Ref
*A. barbadensis* (*A. vera*)	Freeze-dried and ethanol-treated extracts from leaf gel filet	Human cytomegalovirus	Middle stages of infection (DNA synthesis)	[8]
	Glycerin extract from leaves	Herpes simplex virus 1	Inhibited infection in vitro	[9]
	Glycerin extract from leaves Hot glycerin extract from leaf gel	Herpes simplex virus 2	Inhibited infection in vitro; Inhibited pre-entry, entry, post-attachment stages	[9,10] [8,11]
	Commercial freeze-dried powder	Porcine epidemic diarrhea virus	Inhibited replication Inhibited post-entry stages; Protected piglets from intestinal pathology	[12]
	Distilled water precipitate	Murine norovirus 1	Virucidal effects	[13]
*A. secundiflora*	Distilled water leaf exudate	Newcastle disease virus	Reduced disease severity	[11]
*A. hijanensis*	Fractionated leaf homogenate	Newcastle disease virus	Reduced egg infectivity	[14]
	Fractionated leaf homogenate	Influenza A H5N1 virus	Reduced egg infectivity	[14]
	Fractionated leaf homogenate	Egg drop syndrome virus	Reduced egg infectivity	[14]
	Fractionated leaf homogenate	Avian paramyxovirus type 1	Reduced egg infectivity	[14]
*A. arborescens*	Commercial aqueous extract	Human rhinovirus 14	Inhibited infection/production	[15]
	Commercial aqueous extract	Influenza A and B viruses	Inhibited infection/production	[15]
	Commercial aqueous extract	Parainfluenza virus 3	Modest inhibition of infection	[15]
	Commercial aqueous extract	Respiratory syncytial virus	Modest inhibition of infection	[15]
	Commercial aqueous extract	Upper respiratory tract viruses	Reduced recurrence of upper respiratory tract infections in children	[16]

**Table 2:** *Aloe vera* compounds with antiviral activities.

Compound	Virus	Observation/Mode of Action	Ref
Aloe emodin	Herpes simplex virus (HSV)1	Inhibited infection in vitro; disrupted the viral envelope	[9]
	HSV2	Inhibited infection in vitro	[9]
	Varicella zoster virus	Inhibited infection in vitro	[9]
	Pseudorabies virus	Inhibited infection in vitro	[9]
	Influenza A virus	Inhibited infection in vitro	[9]
	Enterovirus 71	Reduced virus production in vitro	[20]
	Japanese encephalitis virus (JEV)	Reduced virus production in vitro; Inhibited JEV NS2B-NS3A protease	[20]
	Severe acute respiratory syndrome coronavirus	Inhibited 3C-like protease	[22]
	Hepatitis B virus (HBV)	Reduced production of HBV S and E antigens; May bind HBV polymerase	[23]
Aloin	Influenza A and B viruses	Inhibited infection in vitro; Improved mouse survival after influenza A H1N1 (PR8) challenge; Accelerated viral clearance in mice	[24]
	HBV	Reduced production of HBV S and E antigens	[23]
	Hemorrhagic septicemia rhabdovirus	Inhibited viral infection in vitro; May disrupt the viral envelope	[25]
Aloesaponarin II	Influenza A virus	Inhibited infection of oseltamivir-susceptible influenza A virus in vitro	[26]
Aloe polysaccharides	Influenza A H1N1 (PR8)	Inhibited virus production in vitro; Protected mice from virus-induced pathology; Caused irregularities in viral particle shape	[27]
Acemannan	Human immunodeficiency virus	Inhibited viral replication; Synergistic effects with azithymidine in vitro	[28]
	Feline immunodeficiency virus	Stable clinical states	[29]
	Feline leukemia virus	Improved clinical signs	[30]

**Table 3:** Immunomodulatory effects of *Aloe vera* components.

Component	Model	Condition/Stimulation	Observed Effects	Ref
Aloe emodin	Rat leukocytes		↓ NK activity, macrophage phagocytosis ↑ IL-1β, TNF-α in leukocytes	[48]
	Mouse macrophages	LPS	↓ *Nos*, *Cox* mRNA ↓ NO, PGE_2_	[49]
	Rat	Arthritis	↓ NO in rat paw edema model	[50]
	Rat	Cerebral occlusion reperfusion	↓ TNF-α in serum of rat model ↓ IL-6, TNF-α, NO in microglial BV2 cells	[51]
	TE-671 cells, HL-CZ cells		**↑** IFN-α, ISG promoters (ISRE, GAS) **↑** ISG (PKR, OAS) mRNA levels	[20]
	MDCK cells	Influenza A virus/ Influenza NS1 protein	↑ Galectin-3, IFN-β, IFNγ ↑ pSTAT1, ISGs (PKR, OAS)	[52]
Aloin	Mouse	Influenza A virus	**↑** CD4^+^, CD8^+^ T cells in lungs **↑** IFN-γ, TNF-α in T cells **↓** TGF-β in T cells	[24]
Aloesin	Rat	Colitis	**↓** Granulocyte infiltration in rat colon **↓** TNF-α, PGE_2_, LTB_4_ in serum **↓***Tnfa*, *Il1b* mRNA	[53]
*A. vera* gel	Human biopsies, Caco-2 cells	Ulcerative colitis	**↓** PGE_2_ in biopsies from patients with active ulcerative colitis **↓** IL-8 in Caco-2 cells	[54]
	Rat	High-fat diet	**↓** TNF-α, TGF-β, and IL-6	[55]
	Macrophages (Primary and cell line)	LPS	**↓** IL-6, IL-8, IL-1β, TNF-α, NLRP3, P2X7 **↓** NF-κB, and MAPK pathway kinases in THP-1 cells and primary macrophages	[56]
	Mouse	Sepsis	**↓** Multiorgan dysfunction **↑** Bacterial clearance, survival **↓** TNF-α, IL-6, IL-1β in serum	[57]
	Rat	Arthritis	**↓** Paw swelling **↓** *Tnfa*, *Cox2* mRNA	[58]
	Mouse	Atopic dermatitis	**↓** Histopathological markers **↓** IFN-γ, IL-4, IL-17A in skin lesions	[59]
	THP-1 cells	LPS	**↓** TNF-α, IL-1β **↑** IL-10	[60]
Acemannan	T cells from human PBMC		**↓** T cell activation and proliferation **↓** IL-2, IL-5, IL-17A	[61]
	T cells from human PBMC	Alloantigen	**↑** Cytotoxic T cell generation **↑** Response to alloantigen	[62]
	Macrophages		**↑** Macrophage activation upon IFN-γ stimulation; **↑** IL-6, TNF-α, NO in macrophages	[63]
	Chicken		**↑** NO in splenocytes and macrophages	[64]
	Mouse		**↑** Mitogenesis of splenocytes **↑** DC maturation and differentiation	[65]

Abbreviations: COX-2: cyclooxygenase 2; DC: dendritic cell; GAS: gamma-activated sequence; IL: interleukin; iNOS: inducible nitric oxide synthase; IFN: interferon; ISG: interferon-stimulated gene; ISRE: IFN stimulation response element (ISRE); NLRP3: OD-, LRR- and pyrin domain-containing protein 3; NF-κB: nuclear factor kappa B; NO: nitric oxide; OAS: 2′,5′-oligoadenylate synthetase; PBMC: peripheral blood mononuclear cells; PGE_2_: prostaglandin E_2_; PKR: protein kinase R; pSTAT1: phosphorylated signal transducer and activator of transcription 1; TGF: transforming growth factor; TNF: tumor necrosis factor. ↑: Increased; ↓: decreased.

**Table 4:** Adjuvanticity of *A. vera* components in viral vaccines.

Virus/Antigen	Adjuvant Form (Administration)	Organism	Observed Effects	Ref
Newcastle disease virus	Acemannan (s.c.)	Chicken	**↑** Protective antibody titers	[79]
Coxsackievirus B3	Acemannan (i.p.)	Mouse	**↑** Antibody titers	[80]
Pandemic AH1N1 antigen, Quadrivalent influenza vaccine	Processed *Aloe* gel (p.o.)	Mouse	**↑** Hemagglutinin and neutralizing antibody titers **↑** Survival after homologous and heterologous challenge	[81]
Myxomatosis virus	*Aloe* polysaccharides (p.o.)	Rabbit	**↑** IgA, IgG, IgM **↑** CD4^+^, CD8^+^ T cells	[82]
Human papillomavirus 16 E7d	*A. vera* gel (p.o.)	Mouse	**↑** IFN-γ, IL-4 (with alum) **↑** IgG2 (with Montanide)	[83]
Quadrivalent influenza vaccine	Processed *Aloe* gel (p.o.)	Human	**↑** Geometric mean titer increase, geometric mean fold increase **↓** Incidence of upper respiratory tract infection	[84]

Abbreviations: IFN: interferon; Ig: immunoglobulin; IL: interleukin; i.p.: intraperitoneal; p.o.: oral; s.c.; subcutaneous. ↑: Increased; ↓: decreased.

## Data Availability

All data is available in this article.
